# Quality improvement in hospitals in the Russian Federation, 2000–2016: a systematic review

**DOI:** 10.1017/S1744133119000252

**Published:** 2020-07

**Authors:** Vasiliy V. Vlassov, Katie Bates, Martin McKee

**Affiliations:** 1 Centre for Health Policy, Higher School of Economics, Moscow, Russian Federation; 2 London School of Hygiene and Tropical Medicine, London, UK

**Keywords:** Quality of care, Russia, systematic review

## Abstract

We reviewed published evidence on quality improvement in hospitals in the Russian Federation since 2000. We used three data sources: MEDLINE, ‘Rossiiskaia Meditsina’ (Central Scientific Medical Library), and elibrary.ru using specific search terms. No language or study design restrictions were imposed. In total, 1717 articles were identified; 51 met the inclusion criteria and were thematically analysed. Russian legislation, government acts and grey literature were sourced to contextualise identified themes. Since 2010, the Federal Ministry of Health has increasingly sought to improve quality of care, providing additional resources and new initiatives across the health system. These include clinical practice guidelines, pay for performance schemes, electronic medical records, more specialist care, paraclinical care, and quality control systems. Quality of care, increasingly a concern of the Russian government, is said to be improving. Yet most initiatives have rarely been evaluated. This reflects the limited capacity for health services research in Russia. It seems likely that the full potential for improvements in quality of care in Russia is still to be realised.

## 1. Introduction

While the Soviet health system had many achievements, it failed to keep pace with developments elsewhere (Field, 1990; Andreev *et al*., 2003). Consequently, in 1991, newly independent Russia inherited large numbers of poorly paid staff, extensive, often obsolete facilities, and shortages of essential supplies, including modern medicines and technology.

The government acted quickly to implement change, introducing a new financing system, based on a combination of taxation and compulsory insurance, while slowly reducing underused capacity, including both facilities and staff. Opening of markets, especially for pharmaceuticals, and removal of Cold War restrictions on importing western high-technology equipment with possible dual civil–military use, led many untested therapies to be abandoned (McKee, 2007). However, progress was limited and, in the mid-2000s, the Russian government launched a large federal programme of investment in health care, although with an emphasis on technology (Shishkin and Vlassov, 2009).

These changes have been reflected in health outcomes. The Healthcare Access and Quality Index (HAQI), developed by the Global Burden of Disease programme, measures deaths from causes amenable to health care, adjusted for age and risk factors (GBD 2016 Healthcare Access and Quality Collaborators, 2018). Measured on a scale from 0 to 100, Russia scored 63.1 in 1990, declining slightly by 2000 to 62.5, before increasing rapidly to reach 75.1 in 2016. The rate of improvement was 1.14 per year between 2000 and 2016, compared with −0.1 year in 1990 to 2000. For comparison, in the 2000–2016 period, the annual rate of increase was 0.95 in Ukraine, to reach 74.6 in 2016. However, there is scope for further improvement. The 2016 figure for Sweden was 95.5 and for the USA it was 88.7.

These figures are supported by other evidence, in particular research on the improvement in life expectancy in Russia that began in 2005, after a period of slow decline over the previous 7 years. This highlighted the major contribution from lower death rates at older ages, especially from cardiovascular diseases (Rechel *et al*., 2013), while another study reported a substantial improvement in access to advanced treatment of myocardial infarction over a similar period (Kontzevaya *et al*., 2017). Although the literature is sparse, there has also been investment in new models of care in other areas, such as obstetric and neonatal care (Shuvalova *et al*., 2015). Many factors may have played a part, including greater coverage, better trained personnel, and access to effective treatments. One possible reason, often overlooked, is improvement in quality linked to rigorous assessment of evidence, development of standards, and measures to implement change and monitor its impact. Soviet science had developed distinctive characteristics from the late 1920s onwards and this, coupled with the difficulties in developing links with western scientists and ideas, led to a growing divergence in approaches. This was illustrated by a failure to employ methods that would be adopted in the West, such as randomised controlled trials and, subsequently, the concept of ‘evidence-based medicine’ as promulgated by, among others, the Cochrane Collaboration, which only took root in Russia in the 1990s (Field, 1990; Rechel *et al*., 2011). In the immediate aftermath of the breakup of the USSR the economic system faced challenges in delivering modern treatment (McKee, 2007) but there has been considerable investment over recent years in health technology assessment. While this is still in its early days (Oortwijn *et al*., 2013; Holownia-Voloskova *et al*., 2018), it is likely to make a growing contribution in the years ahead. There is, however, no contemporary overview of developments in quality of care in Russia, with most evidence difficult to access in the rest of the world as it is written in Russian. Here we report on a systematic review of literature on quality improvement in health care in Russia since 2000 which we hope may improve understanding of these improvements in outcomes over the past two decades.

The review is limited to laws and policies related to hospital care as it forms part of a larger study of the role of hospitals and their governance arrangements in the Russian health system. There has been much less attention in recent reforms to primary care in Russia, as described in a recent study, so this was excluded from the scope of this review (Sheiman *et al*., 2018).

## 2. Methods

Three databases were searched in March 2017, MEDLINE, the electronic catalogue of the Central Scientific Medical Library in Moscow, and elibrary.ru.

### 2.1 Study selection

MEDLINE was searched using the terms: (Quality of Health Care[mh] OR ‘quality of care’ OR ‘care quality’) AND (hospitals[mh] OR hospital medicine[mh]) AND (rus[la] OR russi*[ad]) AND (2000:2017[dp]). The catalogue of the Central Scientific Medical Library ‘Rossiiskaia Meditsina’ was searched using terms: (((((AH больниц*) or (AH стационар*) or (AH больниц*) or (AH больничн*)) and ((AH качеств*) and (AH помощи)))) and (PY BETWEEN ‘2000’,‘2017’)). The elibrary.ru was searched using the query (качеств* AND помощи AND (стационар* OR больниц* OR госпиталь*)) for the years 2000–2017. In addition, 2015–16 editions of the journal ‘Zdravookhranenie Rossiiskoi Federatsii’ were hand searched, as it was the outlet with the most relevant articles. We followed up references in the identified studies where appropriate and supplemented the results with reports from the grey literature and Russian legislation and acts of the Ministry of Health, Government and Federal Fund for Compulsory Medical Insurance issued since 2000. Comments and editorials were excluded, as were conference proceedings and institutional reports, which provided little detailed information, although they dominated the search results from the ‘Rossiiskaia Meditsina’ and e-library databases. No limits as to language or study design were imposed. We registered the protocol with the PROSPERO database of systematic reviews (no. 83892).

### 2.2 Data extraction

**Figure 1.**
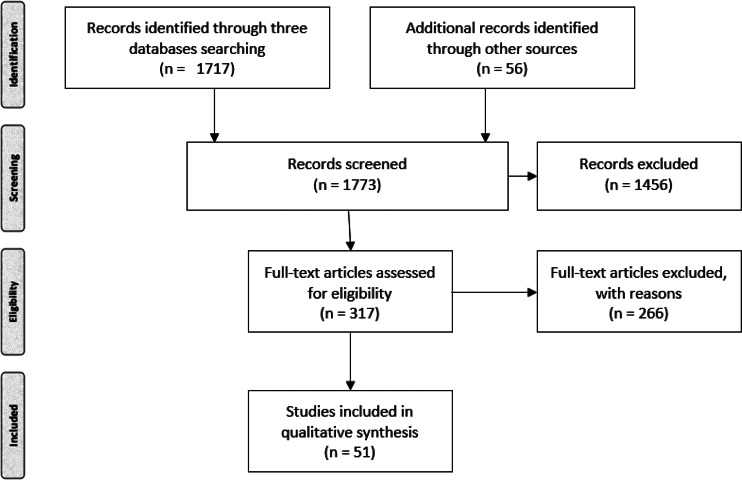
PRISMA chart showing publications selected for the review.

One reviewer (VV) screened all results, initially by title and abstract and, where appropriate, by obtaining full texts. The three databases yielded, respectively, 256, 1341, and 120 records. Eventually, 51 studies were included in the final analysis. The flow chart is presented in Figure 1. The reasons for exclusion were not systematically categorised as most of the publications excluded failed to meet several of the inclusion criteria but common reasons included being only news items, editorials, and commentaries that provided no primary information.

The information was analysed thematically, drawing on a previous review of quality in European health systems, which included countries that had recently transitioned from Soviet health systems (Estonia, Latvia, and Lithuania) as well as others that had similar systems in central and eastern Europe based on the Soviet Semashko system (Legido-Quigley, 2008). This placed initiatives in one of three broad categories, relating to legislation and related policies, organisational reforms, and clinical quality assurance.

Given the limited information in many papers, a risk of bias assessment could not be undertaken meaningfully.

## 3. Results

We present our findings in relation to developments in the Russian health system that, potentially, might have implications for quality, and for each describe the extent to which this has been evaluated.

### 3.1 Legislation and other policies related to quality of care

Prior to 1993, Russian health care facilities were funded mainly by block grants, based on historic budgetary trends. The system underwent a major reform in that year. Working within a national framework, each of Russia's 88 regions implemented a system of Compulsory Health Insurance. This was complemented by a Federal Compulsory Insurance Fund, covering some specialised care and correcting financial imbalances among regions. Some additional funding was provided from federal, regional, and municipal budgets. Providers were paid through a complex mix of budgetary transfers and fee for service payments.

The new system paved the way for greater control over how care was provided, with some funding streams linked to specification of the diagnostic and therapeutic interventions expected to be used for certain groups of patients. The regional medical insurance funds began to use their ability to specify these standards in nascent systems to evaluate quality of care. These initiatives have been credited with creating a climate that encouraged various organisations, at local, regional, and national levels, to develop clinical practice guidelines (CPG) by, initially also called ‘standards’ and later ‘protocols’. A report from Moscow described how some were in widespread use by 2000, having been incorporated into electronic patient management systems (Sizova, 2001). However, there was little co-ordination between these initiatives.

A new national law on technical regulation enacted in 2002 sought to provide greater clarity of responsibilities (Ministry of Health of the Russian Federation, 2002). This removed the right to set standards from the Federal Ministry of Health, giving sole responsibility to non-state bodies. However, the Ministry opposed this move and, by 2006, had reasserted its role to some extent. It fully recovered its role in 2011, in a new federal law on health care (Ministry of Health of the Russian Federation, 2011). The legislators' intention was that it would develop ‘standards’ that would both prescribe the content of care and the resources used. This process was to be led by working groups organised by chief specialists at the Ministry. These standards covered care of a wide range of conditions, in hospital and outpatient care (Movchan, 2013). Providers deviating from these standards could be fined by medical insurance organisations for delivering sub-standard care (Vlassov, 2016).

Having recovered the right to set standards, the Federal Ministry then sought to change the legislation further, linking the standards more closely to financial planning in preparation for the gradual implementation of diagnosis-related group-based payments from 2014. At the same time, it accepted that guidelines on detailed aspects of clinical practice and decision making, which were to be scaled up, would be led by medical professional associations, although under its auspices. In late 2018, the Basic Law on Health Care (#323-FZ) was amended. Now CPG, developed by medical professional organisations, but under the auspices of the Ministry of Health, will become the main source prescribing the content and processes of care, while the previous standards will be used only for economic planning.

In 2015, the Federal Ministry of Health introduced a system of ‘quality control’, based on CPG and standards that include pre-defined quality criteria (Ministry of Health of the Russian Federation, 2015), further revised in 2016 and 2017 (Ministry of Health of the Russian Federation, 2017*a*). The old system had been viewed as a tick box exercise. The new one includes data on delays in treatment and some outcome data. A related initiative was a 2014 federal law allowing for independent evaluation of quality of services by patients and their relatives (Russian Federation, 2014). The Ministry of Health created a public council to support this process but, in practice, assessments provide little information on the quality of clinical care, instead being largely limited to patient satisfaction, which is notoriously low (with only 9% of national sample in 2017 reporting their experience of health care as good, with 37% describing it as acceptable) (VCIOM, 2017).

Unfortunately, although these developments provided many opportunities for evaluation, examining the processes by which guidelines and standards have been developed, the use of evidence, the barriers and facilitators to their implementation, and the extent to which they have changed clinical practice, we were unable to find any rigorous studies that had undertaken the necessary research.

Another policy area where changes may have had implications for quality is the way that physicians are paid. Since late 1990s, some hospitals explored systems of pay for performance to encourage greater productivity and quality of work. By the early 2000s, published reports describing these experiences began to appear (Maliavin *et al*., 2005). However, most were simply descriptive, or before and after studies with no controls, such as an account of a ‘pilot project’ implemented in several regions in 2007–2008, with additional funding from the insurance funds for performance-related pay schemes intended to improve quality of care (Government of the Russian Federation, 2007). Although it is widely believed that these measures were associated with better care, rigorous evidence of actual improvements is lacking and, if they did, the mechanisms by which they did so remain unclear. This is in part due to study design; the regions selected were already better funded than that those not included and differed in other important respects. Furthermore, only very sketchy details are available in the published reports (Zinchuk, 2009).

Despite this limited evidence, performance-related pay has been rolled out widely, with claims, albeit lacking robust evidence, that it has been effective at improving quality (Suslin *et al*., 2014). By 2016, it was estimated that over half of the salaries of both hospital and outpatient physicians was linked to payments based on qualifications, experience, productivity, and quality. Anecdotal evidence suggests that payments related to additional qualifications and quality of care have become much more important than those related to hours of work, although the situation is complicated as the government and regions have sought to improve doctors' earnings, while constrained by legislation on basic pay rates, getting around this by encouraging them to work longer hours. However, again, detailed evidence on performance-related pay is lacking in the published literature.

### 3.2 Organisational reforms

#### 3.2.1 Proprietary quality initiatives and accreditation

Some reports describe experiences in introducing initiatives based on the International Organization for Standardization's (ISO) standards for health care (Kartashov, 2004; Taits *et al*., 2013; Chuhraev *et al*., 2016), but few provide any data on their impact. In one study, members of inpatients' families were surveyed to identify any differences before and after introduction of the quality management system (Lomakin and Shevchenko, 2010), finding some evidence of a positive change. Another study reported increased efficiency of care (Voskanian *et al*., 2004). A more recent initiative is voluntary accreditation with the US Joint Commission International (Roitberg *et al*., 2016*a*) but, as of April 2019, only four private hospitals had been accredited. In 2017, the Federal Ministry of Health encouraged the use of lean quality improvement methods in ambulatory care facilities, but its expansion to the hospitals was not mentioned (Skvortsova, 2017).

#### 3.2.2 Implementation of electronic information systems

In 1996, the Ministry of Health and the Federal Compulsory Insurance Fund introduced a quality control system based on regular sampling of hospital case records (Ministry of Health of the Russian Federation, 1996). While an initial idea to create a system of monitoring based on a nationwide system of computerized patients records was unsuccessful (Siniavskii, 2000), it did encourage moves towards a national health information system. As long ago as the 1980s, a few leading medical institutions in the USSR had experimented with electronic patient records, held on mainframe computers (Vilianskii *et al*., 1987; Stekol'nikov *et al*., 1990). Over time, these systems gave way to new micro-computer-based systems sourced from commercial vendors (Zharkin, 1990). Increased funding of the health care system after 2000 enabled larger hospitals to buy their own computer systems directly from Russian companies (Skliar and Blokhin, 2003). A few reports describe how some institutions have implemented record linkage schemes and undertaken data analysis, with the stated intention of improving the evidence-base and health care they provide (Baranov *et al*., 2016). However, once again, we could find no rigorous evaluations. The ambition of creating a national patient information system, with unified patient records, expressed as recently as 2013, has not yet been realised (Ministry of Health of the Russian Federation, 2017*b*) but in 2019 it was announced that it should be implemented by 2021.

#### 3.2.3 Strengthening specialist care

Between 2005 and 2012, the federal ‘Priority National Project Health’ provided substantial additional resources (Shishkin and Vlassov, 2009). Whilst one of its main stated objectives was to improve access to primary health care, in practice most effort went into strengthening specialist facilities, with an emphasis on purchases of expensive equipment. The new technological possibilities gave rise to systems for regional coordination of specialist care, based on the creation of new centres (Kusch *et al*., 2015). The limited evidence available suggests that this was associated with improvements in performance. Thus, the creation of a specialist centre for inflammatory bowel disease in St. Petersburg was associated with reduced delays in diagnosis and better treatment outcomes. However, the extent to which this was due to additional resources or new approaches to clinical management was unclear (Baranovskii *et al*., 2013). In 2018, a major investment was announced in oncology care, which had been an area of concern, but it is too early to assess what impact this will have.

*Cardiovascular disease and stroke*: Cardiovascular disease and stroke have been given a high priority in Russian health policy, reflecting their extremely high contribution to the total burden of disease (GBD 2016 DALYs and HALE Collaborators, 2017). Thus, it was logical that they would feature prominently in the ‘Priority National Project Health’, leading to increased investment in new equipment. This was associated with a substantial increase in the use of primary revascularisation for acute myocardial infarction across Russia (Erlikh and Gratsianskii, 2009, 2012). This is one of the few major health policy initiatives to have been evaluated in detail, with one study analysing geographic and temporal patterns of expansion of services, demonstrating a reduction in in-patient mortality associated with the improvements in access (Kontzevaya *et al*., 2017). However, that study also showed that progress was very uneven, with some regions, such as those in the North Caucasus, achieving only limited progress, whereas the largest cities, and especially Moscow, had achieved rates of treatment comparable to those seen in Western countries. In Moscow, the creation of a network linking different hospitals offering interventional cardiology in 2013 and 2014 was credited with a large reduction of in-hospital fatality rate for ST-elevation myocardial infarction (Shpektor, 2014). However, some concerns have been voiced about the extent to which improvements in primary care have kept pace with improvements in advanced cardiology techniques (Brazhnik and Zateischikov, 2016).

The management of stroke has also improved progressively after 2000. Several regional centres have introduced thrombolytic treatment and specialized stroke units were opened around the country since 2008. Some major centres now provide endovascular surgery for acute ischemic stroke (Khripun *et al*., 2017). One study in a single hospital compared stroke treatment in a specialized stroke department with that in general neurological wards, finding reduced residual disability at 3 months in the former (Tsurikova *et al*., 2009). However, the study was observational and the two groups may not be comparable. A non-controlled study of use of guidelines for rehabilitation after the cardiovascular surgery among military personnel reported that the duration of rehabilitation could be shortened by 33.1% with improved outcomes (Iudin *et al*., 2014).

*Other specialty care*. We found several reports of studies of maternal and neonatal care, areas where outcomes have improved markedly in recent years. It is believed these improvements are due, at least in part, to better access to quality care for complicated pregnancies (Gadzhiev and Magomedov, 2012), and it has coincided with creation of new perinatal centres as part of the Federal health programme described above. However, evaluation is complicated because of the introduction of a new, expanded, definition of a live birth in 2012. A small randomized control trial found that earlier skin-to-skin contact between mothers and newborns, coupled with allowing newborn babies to be kept in close proximity to their mothers in hospital was associated with better mother–child interactions a year later (Bystrova *et al*., 2009). A comparative study examined the extent to which hospitals are supportive to breast-feeding (based on the international breast friendly hospital initiative) (Abolyan, 2006), finding that hospitals that adopted this model achieved increased rates of breast-feeding and maternal satisfaction, although it found no correlation with health outcomes.

The Federal Ministry of Health has placed a high priority on initiatives to optimize drug usage and blood transfusions, with increased recruitment of clinical pharmacologists in large hospitals (Ministry of Health of the Russian Federation, 1997, 2003). A study in one large hospital described how a series of measures were associated with the reduced use of blood products (Solov'eva *et al*., 2016).

One study by Russian and international researchers reported a high risk of surgical site infections in St. Petersburg (Brown *et al*., 2007), leading to a recommendation to develop a nationwide programme of surveillance of surgical infections, so far not implemented. There are also concerns about increasing resistance of infections to antibiotics in Russian hospitals (Grigorievskaya *et al*., 2017). In 2017, the Ministry of Health proposed a wide ranging plan involving the health, industrial, and agricultural sectors. This would include a requirement for antibiotics to be sold by prescription only, which is not presently the case (Anonymous, 2017).

#### 3.2.4 Improving paraclinical processes

Clinical laboratory services have long been in the forefront of quality assurance in Russian medicine. Acquisition of new automated laboratory equipment, as part of the ‘Priority National Project Health’ speeded up this process (Nikolaev *et al*., 2014), encouraging development of networks of hospitals in Russian cities and regions, a move credited with improving resource use and quality (Ryzhkovskaia and Dzhamedinova, 2016). Subsequently, some laboratory services in larger cities have been outsourced, creating larger facilities with advanced high-throughput systems. One paper from a university hospital in Moscow described benefits from centralizing genetic services (Filippova, 2011).

#### 3.2.5 Clinical quality assurance initiatives

Several studies have been published, most originating from local quality assurance programmes (Tsigankov and Maligin, 2015; Chuhraev *et al*., 2016). In 2015, the Compulsory Health Insurance Fund issued a directive setting out new patient-reported measures of quality (Compulsory Health Insurance Fund, 2015) seeking to integrate patients' ratings of care into the practice of quality control.

Research on patient satisfaction has been undertaken for many years, but mostly using non-standardized and unvalidated approaches, for example in psychiatric care (Tsigankov and Maligin, 2015). One study of hospital care (Loktionova *et al*., 2010) reported that satisfaction among recent inpatients was low, but could be explained by professional qualifications of health workers and aspects of organisation of care. Some other studies did find high levels of patient satisfaction (Gorbunov, 2012). Patient satisfaction surveys were used to identify problems with care quality and, not surprisingly, limited access to specialty care was found a problem (Zhiguleva, 2010). Criteria for quality control for different specialties were developed (Ivanosvskii *et al*., 2011), but we found no reports of their use in the literature.

Detailed methods for quality control within hospitals are not specified in Federal regulations, but it is necessary for some measures to be in place for a hospital to be licensed (in Russia physicians do not obtain licenses but rather medical organizations are licensed). Instead, the systems used to assure quality of health care organizations are prescribed by regional governments. As of 2016, authorities in 59 of the 86 regions of the Russian Federation had issued such regulations (Ivanov, 2016). Their impact has not been reported. Some private health care organizations employ market research methods, such as ‘mystery patients’ and report that this is effective at identifying problems in hospital processes (Roitberg *et al*., 2016*b*). In 2018, the Ministry of Health promulgated a draft regulation on internal quality control, but as of April 2019 the executive order had not been issued.

## 4. Conclusion

There have been many initiatives to improve the quality of care in Russia in the past few decades, involving different approaches, with an overall impression in popular and professional discourse and comments in the peer-reviewed literature that quality of care improved. Yet, on a closer inspection, any research that has been undertaken is very limited. Most is purely descriptive or methodologically weak, in terms of study design and reporting. Most reports are cross-sectional and even before–after studies are rare, with controlled studies almost completely absent. While it is clear that outcomes have improved, with a very few exceptions, any specific influence that major national projects and legislative changes may have had on quality of care remains unknown. This is unfortunate as it constrains the potential to learn about what works in the Russian context. It also highlights the need for a major investment in capacity to undertake health services research.
