# Quality improvement in hospitals in the Russian Federation, 2000–2016: a systematic review – ADDENDUM

**DOI:** 10.1017/S174413311900029X

**Published:** 2020-07

**Authors:** Vasiliy V. Vlassov, Katie Bates, Martin McKee

Keywords: Quality of care; Russia; systematic review; addendum

This article when published neglected to mention one source of financial support, the Basic Research Program at the National Research University Higher School of Economics (HSE). The financial support section of the article should read as follows:

This work was supported by the Wellcome Trust, as part of the International Project on Cardiovascular Disease in Russia (IPCDR) (100217); the Norwegian Ministry of Health; the Norwegian Institute of Public Health; and UiT, The Arctic University of Norway and the Basic Research Program at the National Research University Higher School of Economics (HSE).
